# Fundamental and Essential Knowledge for Pathologists Engaged in the Research and Practice of Immune Checkpoint Inhibitor-Based Cancer Immunotherapy

**DOI:** 10.3389/fonc.2021.679095

**Published:** 2021-07-05

**Authors:** Terufumi Kubo, Tomoyo Shinkawa, Yasuhiro Kikuchi, Kenji Murata, Takayuki Kanaseki, Tomohide Tsukahara, Yoshihiko Hirohashi, Toshihiko Torigoe

**Affiliations:** Department of Pathology, School of Medicine, Sapporo Medical University, Sapporo, Japan

**Keywords:** immune checkpoint inhibitors, pathology, immunohistochemistry, PD-L1, HLA class I

## Abstract

Extensive research over 100 years has demonstrated that tumors can be eliminated by the autologous immune system. Without doubt, immunotherapy is now a standard treatment along with surgery, chemotherapy, and radiotherapy; however, the field of cancer immunotherapy is continuing to develop. The current challenges for the use of immunotherapy are to enhance its clinical efficacy, reduce side effects, and develop predictive biomarkers. Given that histopathological analysis provides molecular and morphological information on humans *in vivo*, its importance will continue to grow. This review article outlines the basic knowledge that is essential for the research and daily practice of immune checkpoint inhibitor-based cancer immunotherapy from the perspective of histopathology.

## Introduction

In the late 19th century, Coley proposed that malignant tumors could be eliminated by means of autologous immunity. Toward this aim, he inoculated cancer patients with heat-inactivated bacteria (1). In the context of immunology, this represents the induction of innate immunity. In the following century, cancer immunology has progressed with repeated cycles of optimism and pessimism. At the end of the 20th century, Boon et al. reported that melanoma-associated antigen-A1 was a specific antigen of malignant melanoma (2). This report provided confidence for the first time that malignant tumors could be specifically eliminated by the patient’s own immune system. However, it took an additional two decades for immunotherapy to become an option for cancer treatment in daily clinical practice. With the advent of immune checkpoint inhibitors (ICIs) in recent years, immunotherapy has been established as the fourth standard cancer treatment method along with surgery, chemotherapy including cytotoxic agents and molecular targeted based agents, and radiotherapy (3). However, the overall response rate of ICIs is only 15%–25% in the approved types of cancer, excluding malignant melanoma (4). However, up to 30% of patients treated with PD-1 and/or CTLA-4 inhibitors experience grade 3 or 4 immune-related adverse events (5). In addition to enhancing its efficacy and reducing harmful adverse effects, the development of relevant biomarkers that can predict the efficacy of immunotherapy is also required. Histopathological analysis is a classic method and the only commonly accessible approach to observe and characterize human diseases including molecular and morphological information *in vivo*. Therefore, the importance of histopathological analysis will continue to grow. This review article outlines the basic knowledge of cancer immunity and the mechanism underlying the effect of ICIs. We also discuss how histopathological analysis can be used to investigate cancer immunity.

## Fundamental Strategy of Cancer Immunotherapy

Before a tumor can become established, the immunosurveillance system eliminates cells that accumulate gene mutations, which are the origin of clinical cancer (6). However, clinically malignant tumors have survived a putative three-step immune-editing mechanism, as we discuss later (7). In other words, a cell with gene mutations needs to evade immunosurveillance in order to develop into a tumor. Therefore, to establish effective immunotherapy, it is necessary to break the tolerance of the immune system to tumor cells. There are two major strategies for cancer immunotherapy: enhancing immunity or reducing immune suppression.

Enhancing anticancer immunity has been a fundamental strategy of cancer immunotherapy for a considerable period of time, from Coley’s bacterial vaccination to cancer vaccines based on antigen-specific peptides or dendritic cells (8). The antigen-specific elimination of tumor cells is the strongest advantage of this approach. However, simply enhancing specific and/or non-specific immunity has not led to clinically relevant cancer immunotherapy because of its low efficacy (9).

In contrast, recently developed ICIs confer an antitumor effect by blocking immune checkpoint-driven immunosuppression. Although the clinical efficacy of ICIs is higher than that of conventional immune enhancement, we are unable to induce cancer-specific immune reactions. Therefore, ICIs often provoke immune-related adverse events (10). ICI-related immune-related adverse events may show characteristic clinical manifestations, which are sometimes different from those of ordinary autoimmune diseases (11). Immune-related adverse events not only deteriorate patients’ quality of life but are also occasionally life-threatening (12, 13).

These two approaches are often described as “pushing the accelerator” and “releasing the brake”, respectively. Notably, these two strategies are not mutually exclusive. Therefore, their combination can be a promising approach for the development of the next generation of cancer immunotherapy.

## Targets of Cancer Immunity

In principle, self-derived antigens are tolerated through thymic selection. Then, how can self-derived tumor cells be targeted by the immune system? Tumor cells possess cancer-specific antigens that are expressed at lower levels by non-tumor tissue. The immunogenicity of a cancer antigen depends on the quantity and quality of the antigen (e.g., the higher its expression level, the higher its antigenicity). However, it has not been fully clarified which factors determine the quality of an antigen. Nevertheless, it is clear that cancer antigen-reactive T cells are not removed as autoantigen-reactive T cells through thymic negative selection (14). Although the classification of cancer antigens has not been standardized, here we simply categorize them into three types: neo-, viral, and self-antigens (15–17). Notably, viral antigens and self-antigens are reproducible among patients, and these can be detected by immunohistochemistry (IHC).

### Neo-Antigens

In the process of cancer development, the accumulation of gene mutations in somatic cells generates proteins with altered structures, which we call neo-antigens. The majority of neo-antigens are not considered to be highly antigenic. However, when increasing numbers of neo-antigens are produced due to the accumulation of a large number of gene mutations, it is more likely that highly antigenic ones will be generated that could serve as specific targets for immunity. Interestingly, the clinical efficacy of ICIs is significantly correlated with the frequency of gene mutations in malignant cells (18), suggesting that ICI-induced cancer immunity targets neo-antigens (19). Because neo-antigens are the product of accidental gene mutations, a specific neo-antigen can principally be applied as a cancer vaccine in a single case. Independent studies in the US and Europe used gene sequencing of tumor tissues to identify putative highly immunogenic neo-antigens, the inoculation of which prevented the recurrence of melanoma (20, 21). Together with the development of gene sequencing technology and sophisticated estimation algorithms for the identification of immunogenic neo-antigens, such personalized treatment may become prevalent in the future (22).

In addition to “ordinary” neo-antigens generated *via* gene mutation, reproducible neo-antigens are attractive targets for next-generation cancer immunotherapy. Because reproducible neo-antigens can be inventory-shared, they can be applied for vaccination therapy or adoptive cell therapy for a considerable number of patients. There are three candidates for inventory-shared neo-antigens: spliced peptides, hotspot mutations, and antigens derived from cancer-specific aberrant post-translational modifications. Proteins are decomposed into peptides by the proteasome and then recombined into spliced peptides (23, 24). Although they do not depend on gene mutations, spliced peptides differ from the original amino acid sequence and can be neo-antigens in a broader sense. Although the generation of spliced peptides often occurs in non-neoplastic cells, especially in the endocrine system, tumor cells also possess them (25). Hotspot mutations often generate diverse mutation-derived neo-antigens. Indeed, hotspot mutations in tumor protein p53 (*TP53*) and isocitrate dehydrogenase 1 (*IDH1*) were reported to generate antigenic peptides in ovarian cancer and glioma, respectively (26, 27). In addition, the identification of hotspot mutation-derived neo-antigens and their application for tailored neo-antigen therapy has become reality (28). Regarding antigens derived from cancer-specific aberrant post-translational modifications, protein phosphorylation can alter the structure of self-peptides to generate tumor-specific epitopes (29–31). The functional relevance and efficient detection of these reproducible neo-antigens are under investigation.

### Viral Antigens

Viral infection is an important factor in the development of cancer. In viral infection-related cancer, viral antigens can be targeted by the immune system. Carcinoma of the uterine cervix is a representative viral infection-related cancer in which the human papilloma virus is critically involved in carcinogenesis. Autologous immunity can target human papilloma virus-derived E6 and E7 proteins, which is the only clinically applied prophylactic anti-cancer vaccine (32, 33). In addition, ICIs are reported to induce an excellent clinical anti-tumor response to Epstein-Barr virus-related malignant tumors, including a subset of gastric cancer and natural killer/T cell lymphoma (34–36). Clinical trials of ICIs in some other types of virus-associated carcinoma are in progress (37). In addition, recent studies have revealed that sequences derived from human endogenous retroviruses, which are remnants of retroviruses integrated into the human genome, can be associated with the clinical response to programmed death-1 (PD-1) blockade in cancer immunotherapy (38, 39).

### Self-Antigens

Malignant cells often over-express apoptosis-inhibiting or cell cycle-regulating molecules in comparison with non-tumor cells. Cancer immunotherapy has applied these over-expressed antigens as specific targets for a long period of time. A strength of this approach is that this type of cancer antigen is highly involved in the survival of malignant cells. Therefore, acquired gene mutations that disable the immune escape effect of these proteins render tumor cells non-viable. We reported that immunization with a survivin-derived peptide, an apoptosis-inhibiting molecule, conferred an immune response in some types of cancer *in vitro* and *in vivo* (40, 41). However, survivin peptide vaccination did not prolong survival in patients with advanced pancreatic adenocarcinoma in a phase II clinical trial (42). None of the other cancer vaccination therapies targeting this type of antigen has been clinically applied. Because this kind of antigen is also expressed at a low level in normal tissue, they tend to show less antigenicity due to immune tolerance.

Cancer-testis antigens (CTAs), which are also included as self-antigens, are thought to be more immunogenic than over-expressed antigens. They are expressed only in the testis and cancer cells. Although the transcriptional expression of CTAs was reported in the thymic medullary epithelium, negative selection for CTAs and consequent immune tolerance was not proven (43). Theoretically, given that the testis is an immune-privileged site due to the lack of human leukocyte antigen (HLA) class I molecules and the presence of the blood-testis barrier, these antigens in cancer cells can only be targeted by immunity. In recent years, some CTAs, which are involved in spermatogenesis, were shown to be highly and specifically expressed in human cancer stem-like cells/cancer-initiating cells of solid tumors. These cancer stem-like cell/cancer-initiating cell-specific antigens induced a strong immune response, suggesting their potential usefulness for immunotherapy specifically targeting cancer stem-like cells/cancer-initiating cells (44).

A recently published study revealed that malignant melanoma tissue harbors numerous tumor-infiltrating lymphocytes, which are self-antigen cognitive (45). Significantly, antigen spreading, a cardinal process for effective cancer immunotherapy, can potentiate not only neo-antigens but also self-antigens during the killing of tumor cells (46–48). Although inoculation with self-antigens alone does not induce a satisfactory immune reaction, combination therapy with additional ICIs may contribute to disease control (49). The evaluation of self-antigens may become increasingly important toward the realization of a persistent anti-tumor effect.

## Process of the Immune Reaction to Tumor Cells and ICIs

**Figure 1:**
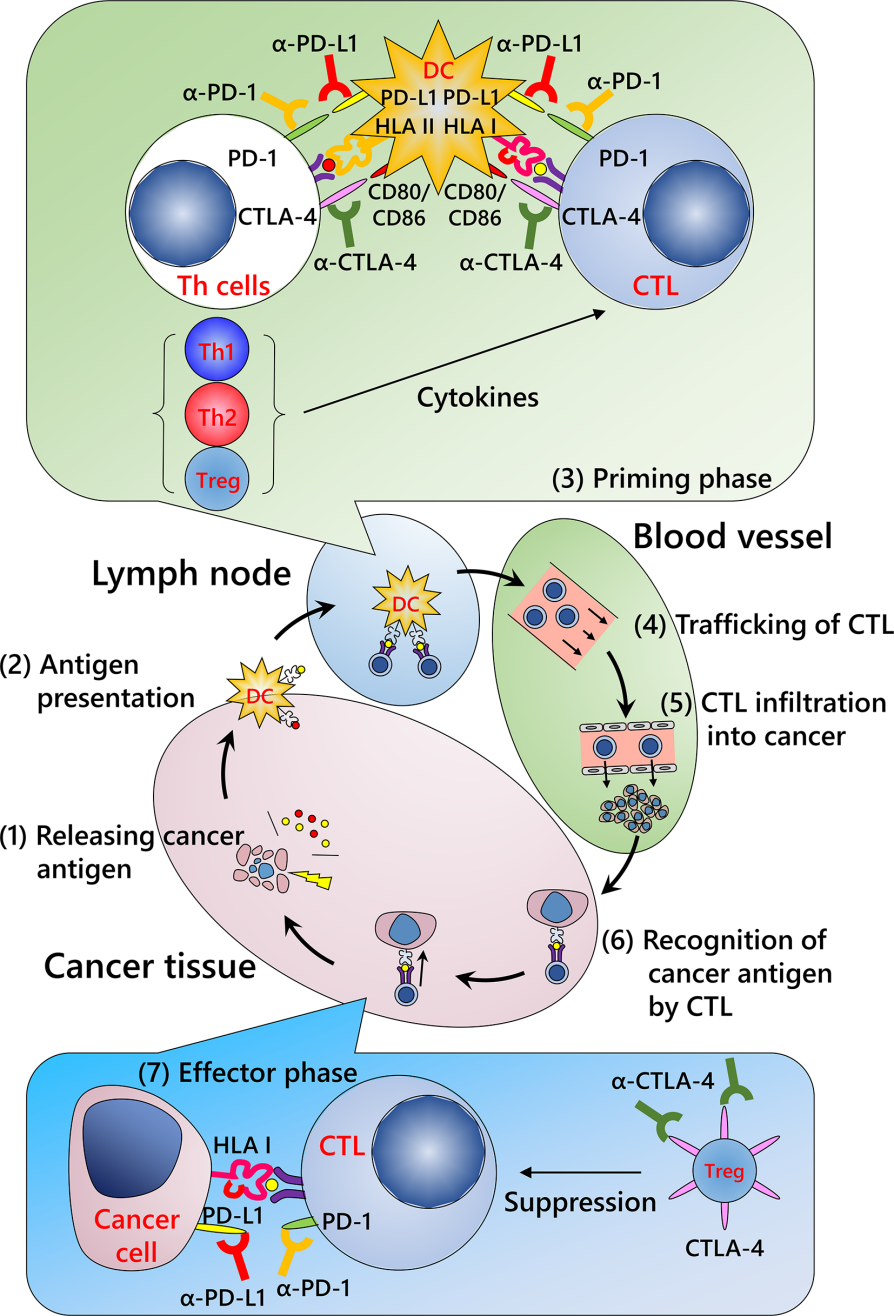
Cancer immunity cycle and mechanism of immune checkpoint inhibitors. (Step 1) Release of cancer antigens from injured tumor cells; (Step 2) uptake of cancer antigens by dendritic cells and antigen presentation; (Step 3) priming phase (T cell activation); (Step 4) migration of cytotoxic T lymphocytes (CTLs) into the tumor; (Step 5) infiltration of CTLs into the tumor; (Step 6) recognition of cancer antigens presented by HLA class I molecules of the tumor cells; and (Step 7) effector phase (destruction of tumor cells). Repeated cycles of the cancer immunity system can eliminate a tumor. Modified from Chen et al. (50) CTLA-4, cytotoxic T-lymphocyte-associated protein 4; DC, dendritic cell; HLA I, human leukocyte antigen class I molecules; HLA II, human leukocyte antigen class II molecules; PD-1, programmed death-1; PD-L1, programmed death ligand 1; Th1, type 1 helper T cell; Th2, type 2 helper T cell; Treg, regulatory T cell.

Cancer immunity involves various types of immune cells such as lymphocytes, innate lymphoid cells including natural killer cells, monocytes/macrophages, and granulocytes. Although several immune cells that can exhibit cytotoxic activity have been reported, including natural killer and T cells, a definite antitumor function in human tumor immunity has been described only for CD8-positive cytotoxic T lymphocytes (CTLs). For immune cells to eliminate malignant cells, it is necessary to complete a series of several functional stepwise events described as the “cancer immunity cycle” (**Figure 1**) (50): release of cancer antigens from injured tumor cells (step 1); uptake of cancer antigens by dendritic cells and antigen presentation (step 2); priming phase (T cell activation; step 3), migration of CTLs to tumor site (step 4); infiltration of CTLs into tumor tissue (step 5); recognition of cancer antigens presented by the HLA class I molecules of tumor cells (step 6); and effector phase (destruction of tumor cells; step 7). Dysregulation of even a single phase stops the whole cycle, resulting in the failure of cancer immunity. Of these steps, the currently available ICIs act in the priming and effector phases.

### Priming Phase

This process occurs primarily in lymph nodes and/or putative tertiary lymphoid structures close to the tumor (51). Tumor antigens from disrupted tumors are taken up by dendritic cells and presented by HLA class I and class II molecules. T cells are activated and proliferate once the following three signals are achieved: T cell receptor recognition of the corresponding antigen peptide-HLA molecule complex (first signal), signaling from the co-stimulatory molecule (second signal), and stimulation by the relevant cytokines (third signal). In this phase, ICIs inhibit co-inhibitory molecules, including PD-1 axis and cytotoxic T-lymphocyte-associated protein 4 (CTLA-4), that repress the second signal.

### Effector Phase

**Figure 2:**
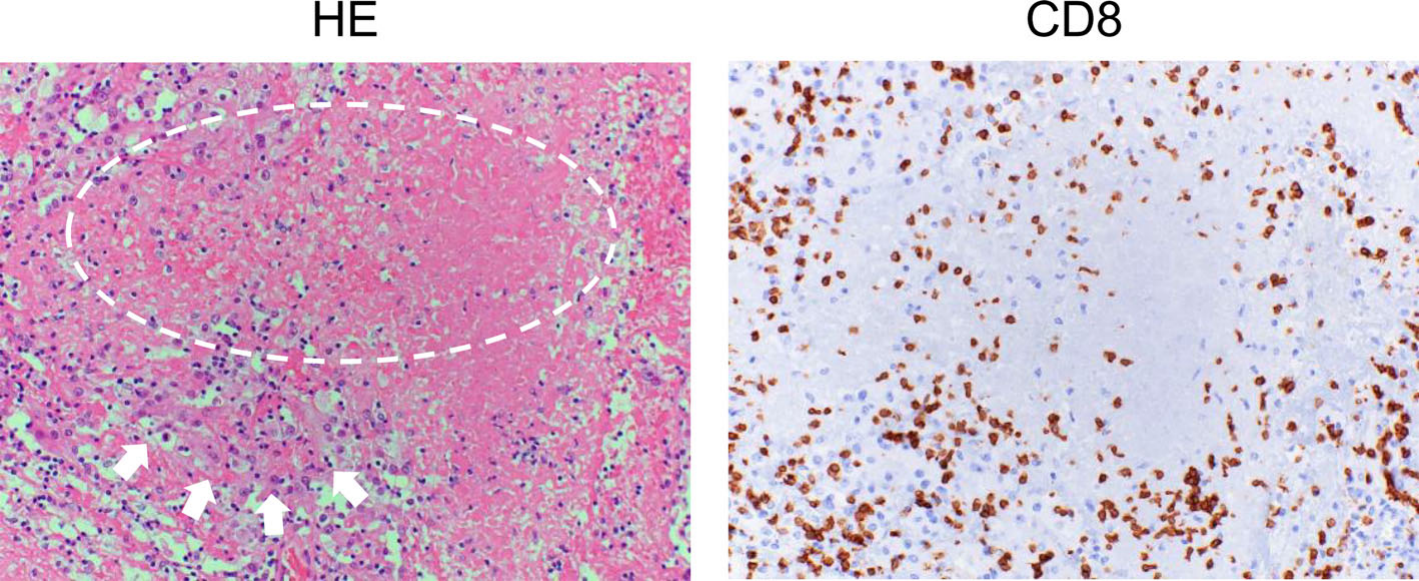
Histology of renal cell carcinoma after the administration of immune checkpoint inhibitors. Left: Hematoxylin and eosin (HE) staining of a case of renal cell carcinoma (arrows) after ipilimumab and nivolumab therapy. Typical morphological changes in apoptosis such as shrinkage of the cell and/or fragmentation into apoptotic bodies are not seen (circle). Right: Immunohistochemical staining for CD8 in a serial section. Numerous CD8-positive cells have infiltrated the tumor.

After the priming phase, activated cancer antigen-specific CTLs migrate and infiltrate into tumor tissue. CTLs recognize cancer antigens presented by HLA class I molecules in the tumor cells and kill the cells. Notably, although it has been considered that CTLs induce apoptosis in tumor cells, these cells do not appear to be apoptotic but rather necrotic in morphological observations (**Figure 2**) (52). In addition, although classic apoptosis is not supposed to evoke inflammation, immunogenic cell death is required to promote the cancer immunity cycle. Indeed, CTL-induced immunogenic cell death has been reported (53). Alternatively, ICIs may evoke secondary necrosis, which is an autolytic process of cell disintegration with the release of cell components when the remnants of apoptotic cells are not cleared by scavenger cells (54, 55). Together with uncovering the mechanism of CTL-induced tumor cell death, it is important to investigate the significance of alarmin proteins as a danger signal released from the destroyed tumor cells in the context of the cancer immunity cycle (56).

In this phase, cancer cells express programmed death ligand 1 (PD-L1) and/or PD-L2, which dampen anti-cancer immunity by interacting with PD-1 on CTLs. Anti-PD-1/PD-L1 antibodies inhibit this reaction. However, anti-CTLA-4 antibody was not considered to have a function in this phase. Nevertheless, the most prevalent anti-CTLA-4 antibody, ipilimumab (IgG1 type), eliminates CTLA-4-expressing regulatory T cells by means of antibody-dependent cellular cytotoxicity (57). Antibody-dependent cellular cytotoxicity activity may increase anti-cancer immunity and unfavorable adverse effects. Tremelimumab, another anti-CTLA-4 antibody (IgG2 type) shows lower antibody-dependent cellular cytotoxicity activity. Compared with ipilimumab, the clinical utility of tremelimumab has not been established. An early study investigating the utility of tremelimumab for malignant melanoma was disappointing, potentially implying the functional significance of anti-CTLA-4 antibody-dependent cellular cytotoxicity activity in the effector phase (58). In addition to these molecules, research and clinical trials have been conducted to investigate whether targeting other types of inhibitory receptors, including lymphocyte activation gene 3 (LAG3), T cell membrane protein 3 (TIM3), and T cell immunoglobulin and ITIM domains (TIGIT), might enhance the efficacy of cancer immunotherapy (59–62).

## Immune Editing and Immune Escape

In our current understanding, the immune system continually detects tumor antigens and eliminates mutant cells regardless of treatment. In the mid-1960s, Bernet named this mechanism “immune surveillance”. However, tumor cells evade immune surveillance by immune editing, which consists of three phases (7). The first is the “elimination phase”, which is the stage where the immune surveillance mechanism works. If elimination fails, it progresses to the “equilibrium phase”, which is the antagonistic state of immune surveillance and immune escape of malignant cells. The final phase is the “escape phase”, in which cells that have accumulated mutations to escape from immunity start to proliferate. Most clinically apparent tumors are at this stage. ICIs can only partially block the immune escape mechanisms in the priming and effector phases. In other words, the reason that many patients do not respond to ICIs is largely attributed to the interruption of the cancer immunity cycle at a certain phase. There are two major patterns of cancer immune escape.

The first is the decreased immunogenicity of malignant cells. As in Darwin’s Theory of Evolution, malignant cells with high adaptation to their environment survive and proliferate. In the context of cancer immunity, a mutant cell with high antigenicity may be eliminated by the immune system. In contrast, cells with low antigenicity can survive.

The second is the cancer cell-mediated reconstruction of the local immune microenvironment. If the expression of co-stimulatory or co-inhibitory molecules is eliminated or increased in tumor cells, respectively, CTLs cannot efficiently eliminate these cells. PD-L1 over-expression on tumor cells is a representative cancer immune escape mechanism. Alternatively, tumor cells and surrounding stromal cells may produce immunosuppressive cytokines such as tumor growth factor-β (63, 64). In addition, tumor cells control the migration, maturation, and/or cytokine production of stromal fibroblasts and/or various immune cells, which leads to the generation of a cancer immune microenvironment with decreased antitumor immunity. For example, the infiltration of regulatory T cells and myeloid-derived suppressor cells into tumors is reported to suppress anti-cancer immunity, resulting in a poor prognosis (65, 66). In contrast, CD8-positive lymphocyte infiltration is often observed only in tumor-associated fibroblastic lesions, but not in tumor cells. In such a setting, CTLs cannot recognize and eliminate tumor cells, which can be the result of a deviated immune cell homing process (67). An increasing number of studies have reported that the manipulation of chemokine-mediated immune cell trafficking ameliorates effector cell infiltration into tumors (68–70).

## Predictive Markers of ICI Efficacy by Histopathological Analysis

ICIs are expensive and can become a socioeconomic burden, and thus relevant biomarkers are urgently required that can distinguish whether an antibody drug would be effective in each patient (71). In our current understanding, there are common denominators that are recognized as potential prediction markers for estimating the efficacy of anti-PD-1/PD-L1 antibodies (72). The first is whether cancer cells express PD-L1, which may mean that tumor cells have escaped from cancer immunity by utilizing the PD-1/PD-L1 axis. The second is whether CD8-positive cells have infiltrated the tumor. The third is how many gene mutations tumor cells have. Strictly speaking, whole exome sequencing is required to estimate mutational burden; however, the detection of defects in DNA mismatch repair proteins (dMMRs) or microsatellite instability can be used as a surrogate marker for mutational burden (73, 74). In addition, the appropriate expression of HLA class I molecules on tumor cells is a prerequisite for CTL-based cancer immunotherapy. Notably, all of these factors can be investigated using formalin-fixed paraffin-embedded (FFPE) specimens and histopathological analysis. The combination of these factors would provide further reliable predictions of treatment efficacy. **Table 1** lists commercially available antibodies for other markers that can be used for the investigation of FFPE specimens. However, the problem is that tumor cells are heterogeneous; therefore, small specimens obtained by biopsy do not always represent the majority of the lesion.

**Table 1:** Representative antibodies for immunohistochemical investigation of human cancer immunity.

Target molecule	Clone	Animal	Localization	Biological function
β2m	D8P1H	Rb	Membrane	Antigen presentation
CD3	- (polyclonal)	Rb	Membrane	Pan T cell marker
CD4	1F6/EPR6855	Ms/Rb	Membrane	Helper T cell marker
CD8	4B11	Ms	Membrane	CTL marker
CD20	L26	Ms	Membrane	Pan B cell marker
CD56	1B6	Ms	Membrane	NK cell marker
CD163	10D6	Ms	Membrane	M2 macrophage
FOXP3	236A/E7/SP97	Ms/Rb	Nuclear	Treg cell marker
Granzyme B	GrB-7	Ms	Cytoplasm	Cytotoxic granule
HLA class I (A, B, Cw)	EMR8-5	Ms	Membrane	Antigen presentation
HLA class I (B, Cw)	HC10	Ms	Membrane	Antigen presentation
HLA class I (A)	HCA2	Ms	Membrane	Antigen presentation
HLA class II (DR)	TAL 1B5	Ms	Membrane	Antigen presentation
IDO-1	SP260	Rb	Cytoplasm	Inducing the immunosuppressive activity of Treg cells
MLH1	ES05	Ms	Nuclear	DNA mismatch repair protein
MSH2	FE11	Ms	Nuclear	DNA mismatch repair protein
MSH6	EP49	Rb	Nuclear	DNA mismatch repair protein
PD-1	EH33/NAT105	Ms/Ms	Membrane	Activated or exhausted T cell marker
PD-L1	E1L3N//28-8/SP142/SP263/22C3	Rb/Rb/Rb/Rb/Ms	Membrane	Immune checkpoint molecule
PMS2	EP51	Rb	Nuclear	DNA mismatch repair protein
Tapasin	TO-3	Ms	Cytoplasm	Required for antigen presentation on HLA class I
TIA-1	TIA-1	Ms	Cytoplasm	Apoptosis-promoting protein

Antibodies listed above are used in our laboratory. Detailed protocols are provided by each manufacturer. Ms, mouse; Rb, rabbit; CD, cluster of differentiation; CTL, cytotoxic T-lymphocyte; NK, natural killer; FOXP3, forkhead box protein p3; HLA, human leukocyte antigen; IDO-1, Indoleamine 2,3-dioxygenase-1; MLH1, MutL homolog 1; MSH2, MutS homolog 2; MSH6, MutS homolog 6; PD-1, programmed cell death-1; PD-L1, programmed death-ligand 1; PMS2, postmeiotic segregation increased 2; TIA-1, TIA1 cytotoxic granule associated RNA binding protein.

### IHC for PD-L1

**Figure 3:**
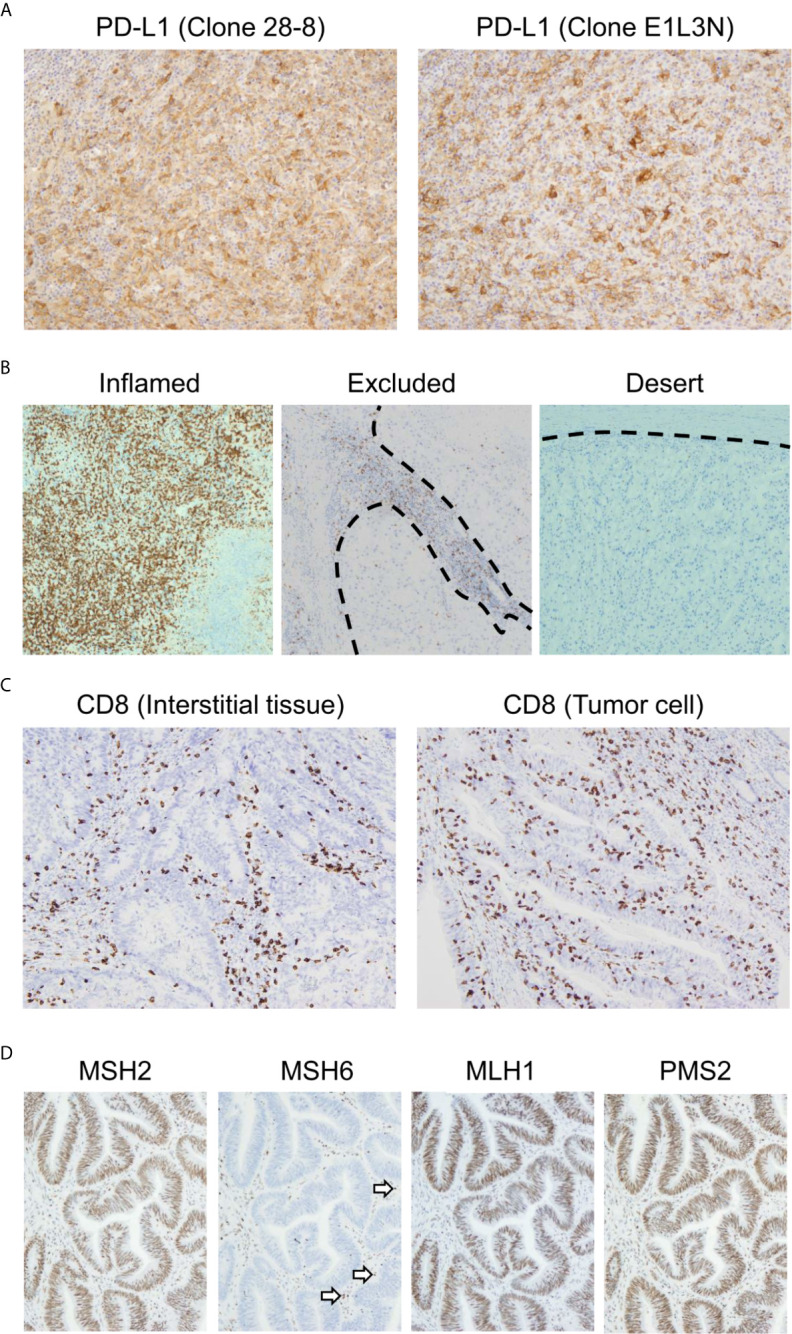
Representative biomarkers estimating the clinical effect of immune checkpoint inhibitors. **(A)** Differential programmed death ligand 1 (PD-L1) staining at the same site on serial sections in a case of melanoma. Left: 28-8 clone; Right: E1L3N clone. **(B)** Three major patterns of CD8-positive cell infiltration. All cases presented here are clear cell renal cell carcinoma. The dotted line indicates the boundary between the tumor and interstitial tissue. **(C)** CD8-positive cell infiltration pattern of colon adenocarcinoma. The maximum density of CD8-positive cell infiltration in these two cases is at the same level. In the left case, CD8-positive cells infiltrated mainly the interstitial tissue. Meanwhile, CD8-positive cells also infiltrated the tumor tissue. **(D)** Immunohistochemistry for MutS protein homolog 2 (MSH2), MutS protein homolog 6 (MSH6), MutL protein homolog 1 (MLH1), and postmeiotic segregation increased 2 (PMS2) in colon adenocarcinoma. MSH2, MLH1, and PMS2 are stained in the nucleus. However, MSH6 staining is lost, indicating DNA mismatch repair protein deficiency. The staining has to be verified with an internal positive control. Note that some of the infiltrated mononuclear cells show nuclear staining (arrows).

Currently, PD-L1 immunostaining with FFPE specimens is performed routinely as a biomarker to estimate the efficacy of anti-PD-1/PD-L1 antibodies. It is worth noting that there are multiple clones of anti-PD-L1 monoclonal antibodies that can be used for IHC. Whereas the 22C3, 28-8, and SP263 clones recognize the extracellular domain of PD-L1, the SP142 and E1L3N clones bind to its intracellular domain (75). PD-L1 staining results differ depending on which antibody clone is used (**Figure 3A**) (76). In particular, the differential recognition domain of each antibody clone affects the results of PD-L1 staining in diffuse large B cell lymphoma. A recent report investigating the interchangeability of PD-L1 IHC concluded that it cannot be simplified (76). Therefore, the appropriate protocol for evaluating PD-L1 expression differs according to the type of cancer to be analyzed.

The interpretation of PD-L1 IHC results is also important. In non-small cell lung cancer, pembrolizumab is significantly more effective in PD-L1-positive cases than in PD-L1-negative cases (77). However, a favorable effect of this antibody drug is not guaranteed even in PD-L1-positive cases. In contrast, pembrolizumab is not necessarily ineffective in PD-L1-negative cases. In clear cell renal cell carcinoma, there was no correlation between the therapeutic efficacy of nivolumab on advanced renal cell carcinoma and PD-L1 tumor expression in the CheckMate-025 study (78). In addition, PD-L1 expression in tumor tissue observed by IHC indicates only a part of the immune environment in the effector phase, but not in the priming phase or entire cancer immunity cycle. Therefore, evaluating PD-L1 expression alone is insufficient for a precise estimate of the clinical response of anti-PD-1/PD-L1 antibodies. It is important to know the pathological significance of PD-L1 IHC results. Although it is difficult to determine this by morphological analysis alone, there are four patterns of PD-L1 staining that take the cancer microenvironment into account (79).

#### PD-L1-Positive Expression Induced by Activated CTLs

Interferon-γ (IFNγ) produced by activated CTLs infiltrating a tumor induces PD-L1 expression in the tumor cells. Consequently, CTLs are inactivated through the PD-1 signaling pathway. This phenomenon is called adaptive immune resistance. In histological analysis, a large number of CD8-positive cells should infiltrate a PD-L1-positive tumor. However, stromal macrophages or other types of immune cells can also express PD-L1. Thus, it is necessary to distinguish whether PD-L1-positive cells are tumor cells or stromal immune cells to predict a therapeutic response, which is sometimes challenging and depends on the cancer type and antibody drug (80–82). For example, when investigating the indication for pembrolizumab in non-small lung carcinoma, pathologists should evaluate PD-L1 expression in cancer cells with the 22C3 clone (77). However, when investigating the indication for atezolizumab in triple-negative breast cancer, pathologists should evaluate PD-L1 expression in infiltrated immune cells with the SP142 clone (83, 84). As a further complication, PD-L1 expression is not important for the application of atezolizumab for non-small cell lung carcinoma after platinum-based chemotherapy (85).

#### PD-L1-Positive Expression Independent of CTLs

PD-L1 over-expression is induced by gene mutation of tumor cells and oncogene activation. This CTL-independent PD-L1 expression is called innate immune resistance. To date, PD-L1 over-expression has been reported in adult T-cell leukemia/lymphoma, in which transcripts are stabilized by disruption of the 3′-untranslated region (86). PD-L1 is over-expressed in Hodgkin’s lymphoma by the amplification of chromosome 9 (87). A similar mechanism has been confirmed in solid tumors at a low frequency. In addition, chemotherapeutic agents can induce PD-L1 expression (88, 89). However, the functional significance of CTL-independent PD-L1 expression has not been established.

#### PD-L1-Negative Expression Due to a Lack of CTL Infiltration

Immune cell trafficking is determined by chemokines and cell adhesion molecules produced by tumor cells and stromal cells, including vascular endothelial cells and fibroblasts (90). Tumor tissue without PD-L1 expression and T cell infiltration is called an “immune desert”, for which administration of anti-PD-1/PD-L1 inhibitor is not expected to produce a response (91). However, the combination of anti-PD-1/PD-L1 inhibitor with an anti-CTLA-4 antibody and some chemotherapeutic agents, which can induce immunogenic cell death, may initiate an effective cancer immunity cycle. Subsequently, CTLs can infiltrate tumor tissue and PD-1 inhibitors can exert an effect through the mechanism described above (92).

#### PD-L1-Negative Expression Due to Gene Mutation

Even when a tumor is profoundly infiltrated with CTLs, PD-L1 expression can be inhibited due to gene mutation such as in the interferon receptor JAK pathway because these mutations prevent IFNγ signal transduction (93). This type of escape has been reported in recurrent cases after anti-PD-1 antibody use (94). In this case, tumor cells are considered to escape from immunity *via* a non-PD-1/PD-L1 axis.

### CD8 and Cytotoxic or Exhaustion Markers

Because PD-L1 expression reflects only a small part of the tumor microenvironment, it can provide a limited prediction of the efficacy of anti-PD-1/PD-L1 treatment. To analyze the tumor microenvironment more precisely, it is necessary to analyze not only tumor cells but also immune cells. The investigation of CD8-positive lymphocytes, which are nearly equal to CTLs, by IHC is the most accessible method for detecting CTLs (95). There are three major patterns of CTL infiltration: inflamed, excluded, and desert (**Figure 3B**) (96, 97). In the inflamed pattern, the tumor harbors numerous CD8-positive cells, whereas they are found only in interstitial tissue in the excluded pattern. In the desert pattern, there are very few CD8-positive cells in the tumor and interstitial tissue. It is especially important to distinguish whether CD8-positive lymphocytes infiltrate the tumor or interstitial tissue. In **Figure 3C**, the maximum density of CD8-positive cell infiltration in these two cases was at the same level. However, the left panel is categorized as the excluded pattern, whereas the right panel represents the inflamed pattern, for which we can expect immunotherapy to be effective. Previous reports have shown the importance of distinguishing these infiltration patterns (40, 98).

**Figure 4:**
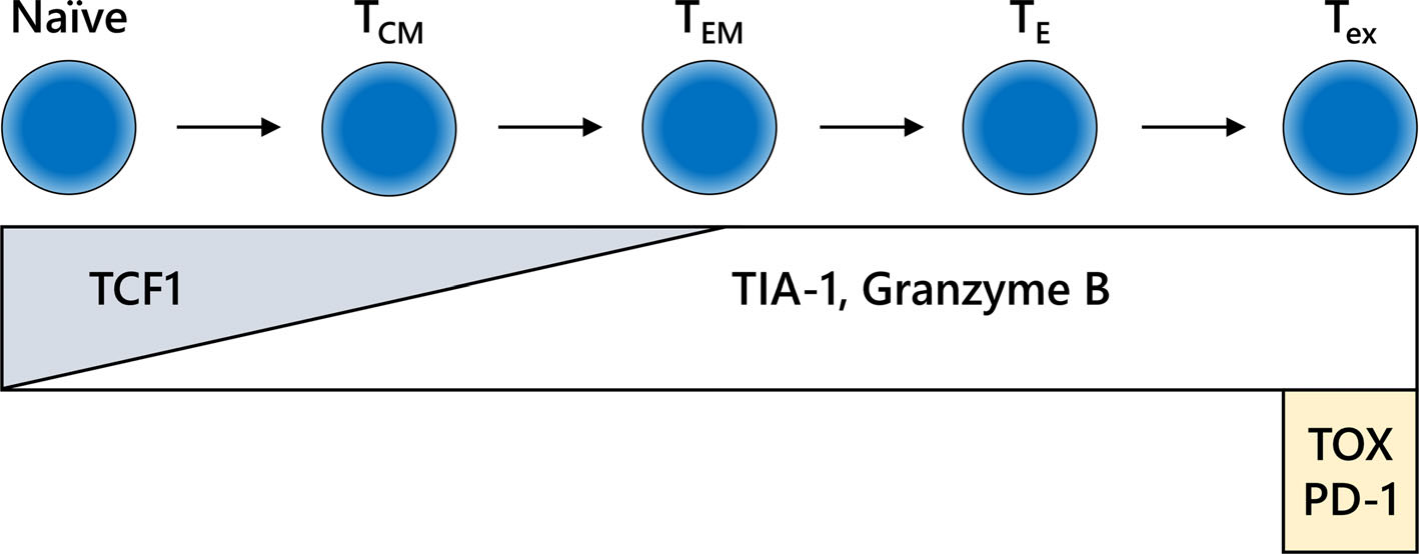
Differentiation fate and immunohistochemistry (IHC) markers for CD8-positive T cells. The behavior of CD8-positive T cells is dependent on the differentiation stage, which is not clearly defined. The schema shown here is extremely simplified. IHC for formalin-fixed paraffin-embedded specimens may reveal the functional relevance of CD8-positive T cells in each stage of differentiation. T_CM_, central memory T cell; T_E_, effector T cell; T_EM_, effector memory T cell; T_ex_, exhausted T cell.

CD8-positive lymphocytes do not consist of a uniform population from the point of view of functional classification. In addition to their location, the functional phenotype or differential status of CD8-positive lymphocytes is an also important information (**Figure 4**). The combination of CD8 with granzyme B and/or TIA-1, which are killing activity-related molecules that are screened frequently in FFPE specimens, may increase reliability (99, 100). In addition, the intratumoral infiltration of transcription factor T cell factor 1 (TCF1)/TCF7-positive CTLs, indicating central memory CD8-positive T cells, is correlated with a positive clinical outcome in melanoma patients (101, 102). Furthermore, potentially useful antibodies detecting IFNγ-inducible molecules, including C-X-C motif chemokine (CXCL) 9, CXCL10, and CXCL11 or IFNγ itself, have been generated (103–105). However, continuous exposure of T cells to antigen induces the deterioration of T cell function, which is called “T cell exhaustion” (106). Exhausted CTLs do not show sufficient antitumor activity in response to inhibition of the PD-1/PD-L1 axis. An increasing number of studies have reported the molecular mechanism and relevant markers of T cell exhaustion. The expression levels of nuclear orphan receptor 4a and thymocyte selection-associated high-mobility group box transcription factors in CD8-positive T cells can be used to determine T cell exhaustion (107–110). Additional examinations of these molecules by IHC might confer further precise evaluation of ICI efficacy.

### Detection of Deficiency of Mismatch Repair Proteins

ICIs are expected to be effective for tumors with a high mutational burden or virus-related tumors. The former includes mutagen-induced tumors such as smoking-associated carcinoma, and tumors with disturbed DNA repair systems. There are several DNA repair systems that maintain the accuracy of DNA replication.

Mismatch repair proteins amend errors of the DNA sequence during DNA replication. Germline mutation of these proteins, which is called Lynch syndrome, significantly increases the lifetime risk of colorectal and/or endometrial carcinoma (111, 112). In addition to approximately 10% of cases with colorectal carcinoma and 30% of cases of endometrial carcinoma, sporadic or germline dMMRs are also found in ovarian, urothelial, gastric, hepatobiliary, and pancreatic carcinoma (113, 114). Pembrolizumab, an anti-PD-1 antibody, has been adapted for the treatment of any type of dMMR cancer. In a famous clinical study, pembrolizumab was shown to be clinically effective in more than 50% of dMMR cancers (115).

In histopathological analysis, four major dMMR proteins, namely, MutL protein homolog 1 (MLH1), MutS protein homolog 2 (MSH2), MutS protein homolog 6 (MSH6), and postmeiotic segregation increased 2 (PMS2), can be examined by IHC (**Figure 3D**). In daily practice, a two-antibody approach with MSH6 and PMS2 is as effective as a four-antibody method (116), because negative staining for MSH6 corresponds to a lack of MSH2 and/or MSH6 proteins because the stability of MSH6 is dependent on MSH2. In the same way, staining for PMS2 covers the protein expression of PMS2 and/or MLH1. Therefore, the loss of dMMR proteins is designated in cases with either the loss of MSH6 or PMS2 staining. The pitfalls and caveats in assessing IHC results for these proteins are described elsewhere (113, 117). Due to the simplicity of this assay, the evaluation of dMMR proteins by IHC is useful for estimating the tumor mutational burden.

### HLA Class I Molecule Expression

**Figure 5:**
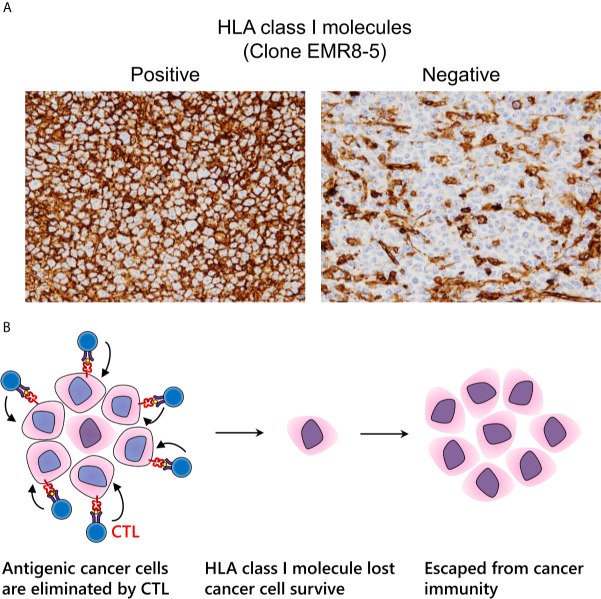
Immune escape *via* loss of human leukocyte antigen (HLA) class I molecules. **(A)** Expression pattern of HLA class I molecules detected by the EMR8-5 monoclonal antibody in diffuse large B cell lymphoma. Left: intact cell boundary staining; Right: loss of staining. **(B)** Adaptive immune escape hypothesis: immunogenic tumor cells are eliminated by cytotoxic T lymphocytes; nevertheless, tumor cells without HLA class I molecules survive and proliferate.

Under immune pressure, tumor cells that no longer express HLA class I molecules can survive due to loss of immunogenicity (**Figure 5A**) (118, 119). Regardless of therapeutic intervention, HLA class I molecules often disappear. We can assess HLA class I molecule expression by IHC examination with FFPE specimens. Notably, it is important for pathologists to evaluate HLA class I molecules on the cell surface, but not in the cytoplasm (120). In surgically resected specimens, a decrease of HLA class I molecules is correlated to a poor prognosis in various types of malignancy, indicating that immune surveillance also inhibits the further growth of an established tumor (118, 121–124). Nearly all current strategies for CTL-mediated immunotherapy cannot theoretically surmount the loss of HLA class I molecules, which is a serious problem for the future of cancer immunotherapy (**Figure 5B**). It is urgently required to establish a relevant scoring system that is reproducible among pathologists. In addition to HLA class I molecules, the proper contribution of the antigen processing and presentation machinery, including β2-microglobulin, transporter 1, ATP-binding cassette subfamily B member (TAP1), TAP2, or tapasin, is required for appropriate cancer antigen presentation. Therefore, dysfunction of these molecules is involved in cancer immune escape (125).

We previously reported that the expression of HLA class I molecules tends to be reduced in dMMR colon cancer (120). We can assume that dMMR tumor cells harboring abundant neo-antigens are naturally exposed to a strong immune reaction, whereas the expression of HLA class I molecules and β2-microglobulin can be inhibited easily, thereby enabling immune-escaped malignant cells to proliferate. We name this phenomenon “adaptive immune escape”. However, surprisingly, in endometrial cancer, this tendency is not the case, potentially because of differences in the immune microenvironment between the two types of carcinoma (126).

## Unresolved Issues and Future Directions of Histopathological Investigation in Cancer Immunology

Although, as noted above, histopathological analysis provides valuable *in vivo* information in humans, a number of issues remain unresolved. Such issues are attributable mainly to the spatiotemporal phenotypic heterogeneity of cancer cells in cancer tissue. Despite its utility and convenience, histopathological investigation provides only a two-dimensional picture of the three-dimensional tumor mass at a given point in time. This raises questions the following questions. How much area needs to be evaluated? Which area should be analyzed? Should it be the core of the tumor, the invasion edge, or both? (127) It is possible that one area harbors a dense group of inflammatory cells, while the other area has far less inflammatory cell infiltration. These points of evaluation vary according to the study. It is still not known how other treatment modalities, including various chemotherapeutic agents and irradiation, influence the cancer immune microenvironment. In addition, there is no established consensus on which timing of biopsy can most accurately predict the clinical efficacy of ICIs. Recent studies have reported positron emission tomography-based monitoring of CD8-positive cell infiltrates in the tumor (128, 129). This technology may provide a promising monitoring tool for investigating specific molecular targets in tumor and/or interstitial cells in the whole cancer lesion. In addition, image analysis involving deep learning methods based on artificial intelligence and neural networks may provide even more accurate evaluation (130). However, it is essential for the development of these technologies to establish an optimal methodology for carrying out histopathological investigation.

## Conclusion

Although cancer immunotherapy is becoming a major standard treatment, we still have many unclear points regarding the detailed mechanism or action of ICIs. In addition, single agent administration is less effective in more than 70% of cases. The risk of serious immune-related adverse events cannot be ignored. Therefore, the development of more effective and highly cancer-specific immunotherapy and the development of reliable biomarkers for optimal treatment selection are important issues for the future. Histopathological analysis by IHC will become progressively more important due to the limitation of accessibility to clinical samples and the daily feasibility of analysis. Furthermore, the recently developed Immunoscore evaluation method of FFPE specimens has provided a prognostic estimation as accurate as that of the tumor, node, metastasis evaluation system (131–134). The evaluation of the immune microenvironment may be required in diagnostic routine in the near future. Immunopathologic research by pathologists, who can form a bridge between clinicians and basic researchers, might lead to the development of better approaches for the understanding and treatment of cancer.

## Author Contributions

Tku and YH wrote the manuscript, developed the ideas, conducted the review of literature, and made the figures. KM, TKa, TTs and TTo has put forward many suggestions in the manuscripts. TS, YK reviewed the manuscript and helped with the editing process, and helped in guiding TKu. All authors contributed to the article and approved the submitted version.

## Funding

This work was supported by the Project for Cancer Research and Therapeutic Evolution (P-CREATE) from the Japan Agency for Medical Research and development (AMED) for T. Torigoe.

## Conflict of Interest

The authors declare that the research was conducted in the absence of any commercial or financial relationships that could be construed as a potential conflict of interest.
